# 

**Published:** 1863-04

**Authors:** 


### CONTENTS.

ART. PAGE

I. On the Nature of Volition. By J. Lockhart Clarke, F.R.S. . 193

II. The Diet of the Insane. By Henry M'Cormac, M.D. . . . 219

III. On Mental and Physical Life in Relation to Time . . . . . 224

IV. The Influence of Material Objects upon the Mind. By J. Alexander Davies . . . . . . . . . . . . . . . . 234

V. A Rare Case of Mania. By Hugh G. Stewart, M.D. . . . 254

VI. The Cotton Famine . . . . . . . . . . . . . . 261

VII. Phrenology and Character. By Edwin Goadby . . . . . 265

VIII. American Asylums for the Insane . . . . . . . . . 286

IX. Hypochondriasis . . . . . . . . . . . . . . . . 300

X. On the Diagnosis and Treatment of Morbid Impulse. By W. Carmichael McIntosh, M.D. . . . . . . . . . . . 305

XI. Sanitary Instruction for the Masses . . . . . . . . . 321

XII. Lunacy on the Stock Exchange . . . . . . . . . . . 329

XIII. Autobiography of the Insane . . . . . . . . . . . . 333

XIV. The Insane in France . . . . . . . . . . . . . . 340

XV. The Medical Evidence of Crime . . . . . . . . . . . 363

XVI. St. Thomas's and Bethlem Hospitals . . . . . . . . . 370

Medico-Legal Trials in Lunacy . . . . . . . . . . . 376

Works Published during the Quarter . . . . . . . . . 390

No. XI. will be published on the 1st of July.

